# International consensus recommendations for the identification and treatment of tuberous sclerosis complex-associated neuropsychiatric disorders (TAND)

**DOI:** 10.1186/s11689-023-09500-1

**Published:** 2023-09-14

**Authors:** Petrus J. de Vries, Tosca-Marie Heunis, Stephanie Vanclooster, Nola Chambers, Stacey Bissell, Anna W. Byars, Jennifer Flinn, Tanjala T. Gipson, Agnies M. van Eeghen, Robert Waltereit, Jamie K. Capal, Sebastián Cukier, Peter E. Davis, Catherine Smith, J. Chris Kingswood, Eva Schoeters, Shoba Srivastava, Megumi Takei, Sugnet Gardner-Lubbe, Aubrey J. Kumm, Darcy A. Krueger, Mustafa Sahin, Liesbeth De Waele, Anna C. Jansen

**Affiliations:** 1 Division of Child and Adolescent Psychiatry, Centre for Autism Research in Africa (CARA), University of Cape Town, 46 Sawkins Road, Rondebosch, Cape Town, 7700 South Africa; 2 Mental Health and Wellbeing Research Group, Department of Public Health, Vrije Universiteit Brussel, Brussels, Belgium; 3 School of Psychology, University of Birmingham, Birmingham, UK; 4 TSC Clinic Cincinnati Children’s Hospital Medical Center, Cincinnati, OH USA; 5 Division of Neurology, Department of Pediatrics, University of Cincinnati College of Medicine, Cincinnati, OH USA; 6 TSC Canada, Mississauga, ON Canada; 7 Department of Pediatrics, University of Tennessee Health Sciences Center, Memphis, TN USA; 8 Le Bonheur Children’s Hospital and Boling Center for Developmental Disabilities, Memphis, TN USA; 9 Emma Children’s Hospital, Amsterdam University Medical Center, Amsterdam, Netherlands; 10 TAND Expert Centre, ‘S Heeren Loo, Hoofddorp, Netherlands; 11 Child and Adolescent Psychiatry, University Medical Center Göttingen, Göttingen, Germany; 12 Department of Neurology, University of North Carolina at Chapel Hill, Chapel Hill, NC USA; 13 Department of Psychopathology and Mental Health, Pedro de Elizalde Hospital, Buenos Aires, Argentina; 14 Department of Neurology, Boston Children’s Hospital, Harvard Medical School, Boston, MA USA; 15 TSC Alliance, Silver Spring, MD USA; 16 Department of Clinical Genetics, St George’s University Hospitals, London, UK; 17 Sussex Renal Unit, The Royal Sussex County Hospital, Brighton, UK; 18 Be-TSC, Mortsel, Belgium; 19 TSCi, Mortsel, Belgium; 20 Society of Parents of Children With Autistic Disorders (SOPAN), Mumbai, India; 21 Japanese Society of Tuberous Sclerosis Complex, Family Network, Tokyo, Japan; 22 MuViSU (Centre for Multi-Dimensional Data Visualisation), Department of Statistics and Actuarial Sciences, Stellenbosch University, Stellenbosch, South Africa; 23 Rosamund Stone Zander Translational Neuroscience Center, Boston Children’s Hospital, Boston, MA USA; 24 Department of Paediatric Neurology, University Hospitals Leuven, Louvain, Belgium; 25 Department of Development and Regeneration, KU Leuven, Louvain, Belgium; 26 Department of Pediatrics, Koningin Mathilde Moeder-en Kindcentrum, Antwerp University Hospital, Antwerp, Belgium; 27 Department of Translational Neurosciences, University of Antwerp, Antwerp, Belgium

**Keywords:** Tuberous sclerosis complex, TAND, Rare genetic disorders, Consensus recommendations, Neurodevelopmental disability, Mental health, Education

## Abstract

**Background:**

Tuberous sclerosis complex (TSC) is associated with a wide range of physical manifestations for which international clinical recommendations for diagnosis and management have been established. TSC is, however, also associated with a wide range of TSC-Associated Neuropsychiatric Disorders (TAND) that are typically under-identified and under-treated yet associated with a profound burden of disease. The contemporary evidence base for the identification and treatment of TAND is much more limited and, to date, consensus recommendations for the diagnosis and management of TAND have also been limited and non-specific.

**Methods:**

The TANDem project was launched with an international, interdisciplinary, and participatory consortium of 24 individuals, including TSC family representatives, from all World Health Organization (WHO) regions but one. One of the aims of the TANDem project was to generate consensus recommendations for the identification and treatment of TAND. At the time of this project, no internationally adopted standard methodology and methodological checklists existed for the generation of clinical practice recommendations. We therefore developed our own systematic procedure for evidence review and consensus-building to generate evidence-informed consensus recommendations of relevance to the global TSC community.

**Results:**

At the heart of the consensus recommendations are ten core principles surrounded by cluster-specific recommendations for each of the seven natural TAND clusters identified in the literature (autism-like, dysregulated behavior, eat/sleep, mood/anxiety, neuropsychological, overactive/impulsive, and scholastic) and a set of wraparound psychosocial cluster recommendations. The overarching recommendation is to “screen” for TAND at least annually, to “act” using appropriate next steps for evaluation and treatment, and to “repeat” the process to ensure early identification and early intervention with the most appropriate biological, psychological, and social evidence-informed approaches to support individuals with TSC and their families.

**Conclusions:**

The consensus recommendations should provide a systematic framework to approach the identification and treatment of TAND for health, educational, social care teams and families who live with TSC. To ensure global dissemination and implementation of these recommendations, partnerships with the international TSC community will be important. One of these steps will include the generation of a “TAND toolkit” of “what to seek” and “what to do” when difficulties are identified in TAND clusters.

## Background

Tuberous sclerosis complex (TSC) is a rare genetic disorder associated with mutations in the *TSC1* or *TSC2* genes, a wide range of physical manifestations, and a highly heterogeneous clinical presentation [1, 2]. TSC is also associated with a broad range of behavioral, psychiatric, intellectual, academic, neuropsychological, scholastic, and psychosocial difficulties [3–5]. Until the 1990s, diagnosis, monitoring, and treatment of TSC tended to be inconsistent and highly variable across the globe. In an attempt to standardize the diagnosis of TSC, a consensus conference was convened in 1998. The meeting led to a simplified and revised set of diagnostic criteria for TSC [6] and was accompanied by recommendations for diagnostic evaluation [7]. The 1998 and 1999 consensus publications provided a structured approach to the physical manifestations of TSC. Given the emerging awareness of the neuropsychiatric manifestations of TSC at the time, the consensus panel also aimed to include information about neuropsychiatric manifestations of TSC, albeit in a limited manner. The recommendations made in relation to “neurodevelopmental testing” suggested “thorough age-appropriate screening for behavioral and neurodevelopmental dysfunction” at the time of diagnosis, and reassessment at school entry. “Periodic” retesting was recommended for older children with “previous test abnormalities,” for those with “abnormal cognitive function or behavior,” and when there was a significant change in behavior. No evaluations were recommended for newly diagnosed adults who appeared not to have any difficulties, and no further evaluations were recommended of those who appeared “normal” or had “stable disabilities” [7].

To provide a more systematic and proactive set of recommendations for the assessment of “cognitive and behavioral problems” in TSC, a meeting was convened in Cambridge, UK, in 2003. The consensus panel, consisting of 20 clinicians, researchers, and family representatives from the USA, UK, and the Netherlands, made two main recommendations [5]. First, to perform a comprehensive evaluation at diagnosis and at key developmental timepoints (infancy, toddler years, pre-school, early school years, middle school years, adolescence, and in young adults). Importantly, this recommendation was aimed at the evaluation of *all* individuals with TSC and not only those with apparent concerns. The panel provided detailed guidelines for developmentally based, comprehensive assessment [8]. The second recommendation was to perform a comprehensive evaluation when sudden or unexpected change or deterioration was observed, mainly to ensure the identification of biological causes of behavioral difficulties, for example growing Subependymal Giant Cell Astrocytoma (SEGA) or poorly controlled seizures. Other than generic comments, no guidelines were provided for intervention. Even though the recommendations were well received in the TSC community, a decade after the publication, fewer than 20% of individuals in the UK with TSC had actually received a comprehensive neuropsychiatric assessment as proposed by the consensus panel, and only about 40% of individuals in a large-scale natural history study of TSC had ever had an evaluation of their intellectual ability [5, 9–11]. These findings suggested a significant “assessment and treatment gap” for these manifestations of TSC [11].

In 2012, an International Consensus Conference was convened which included 79 experts from 14 countries. Expert panels, including a neuropsychiatry panel, made recommendations for diagnosis, monitoring, and treatment of the range of organ systems involved in TSC [12]. At this meeting, the neuropsychiatry panel coined the term “TAND” (TSC-associated neuropsychiatric disorders) as an “umbrella” term for the range of bio-psycho-social difficulties associated with TSC, and to create a “shared language” by describing TAND across 6 “levels” (behavioral, psychiatric, intellectual, academic, neuropsychological, and psychosocial) [5, 12]. In addition to comprehensive assessment at key developmental timepoints and in response to sudden or unexpected change or deterioration in TAND, the panel introduced a new recommendation for *annual screening* of all people with TSC. Some guidance for the intervention of TAND was provided in the consensus guidelines, but in a non-specific manner (e.g., recommending the use of general population evidence-based guidelines for individual manifestations). The 2012 recommendations were updated in 2021, mainly to include consensus recommendations for the use of mTOR inhibitors for physical manifestations of TSC [1]. The TAND-specific recommendations added included the use of screening tools (such as the TAND Checklist), early identification and treatment of TAND manifestations, and psychosocial support to families [1].

Since the 2012 consensus conference and coining of the term “TAND,” a number of new research developments have emerged. These included development and pilot validation of the TAND Checklist (Lifetime version, TAND-L) [5, 13], natural history, and longitudinal studies on the emergence and development of various TAND manifestations [4, 14–17], the identification of “natural TAND clusters” (natural groupings of TAND manifestations) [18–21], and studies on the impact of molecularly targeted treatments using mammalian/mechanistic target of rapamycin (mTOR) inhibitors on TAND, albeit with mixed and unclear results [22–24]. The TAND Checklist was recommended as a tool for screening in the 2021 revised recommendations [1], but no specific recommendations were made for the identification of natural TAND clusters. More importantly, to date, no specific consensus recommendations have been made for the treatment of any aspect of TAND.

In 2019, the TANDem project was launched as an international, interdisciplinary, and participatory project with three main aims [9] (www.tandconsortium.org). The first aim was to develop and validate a self-report and quantified version of the TAND Checklist (referred to as the TAND-SQ Checklist) and to build it into a mobile application (“app”). The second aim of the project was to generate consensus clinical recommendations for the identification and treatment of TAND as the foundation for a “TAND toolkit” of evidence-informed consensus information and self-help tips to be built into the app. The third aim was to establish a global TAND network through research capacity-building and a range of impact activities [9]. The focus of this paper is on one of the specific objectives of the TANDem project—the generation of consensus recommendations for the identification and treatment of TAND.

There is an ongoing discourse in the literature about the need to balance “evidence” and “expert consensus” in the generation of clinical practice recommendations [25]. Historically, many authors described their recommendations either as “evidence-based” or as “consensus-based.” In the TANDem project, we acknowledged at the outset that the evidence base for the identification and treatment of TAND may be very limited and/or of poor quality and that we may need to include the opinions of TSC experts as well as evidence from outside TSC-specific literature. However, we wanted to examine *all* existing evidence from the TSC literature to ensure an unbiased evidence-informed approach to our consensus-building, thus balancing “evidence” and “expert consensus.” We also recognized that access to identification and treatment of TAND and resources to support these actions may be highly variable across the globe. This led us to prioritize higher-level conceptual rather than very detailed recommendations in our consensus-building process.

Here we describe the process of evidence-informed consensus generation and present a set of core principles and cluster-based recommendations for the identification and treatment of TSC-associated neuropsychiatric disorders (TAND).

## Methods

**Fig. 1:**
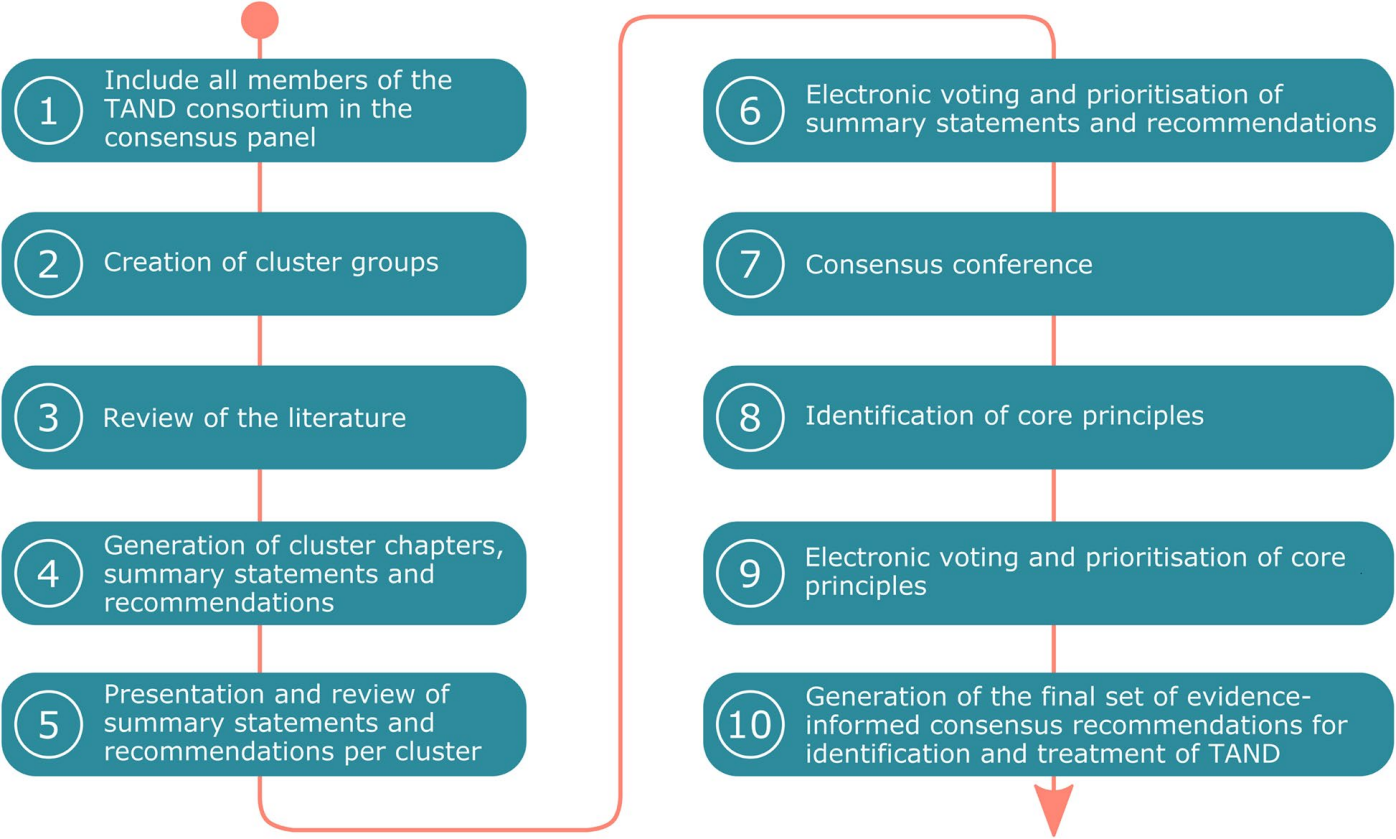
Flowchart showing the process for evidence-evaluation and consensus-building in this study

There are at present no formal guidelines for the reporting of consensus-based methods in biomedical research or clinical practice, but encouragingly, a team of researchers has initiated a process to generate what will be known as the ACCORD guidelines [26]. In the absence of a standard methodology and methodological checklist, we created a systematic procedure for evidence generation, review, and consensus-building as outlined in Fig. 1. The majority of activities took place online given the travel restrictions due to the COVID-19 period in the 2020–2022 timeframe of the study.

### Step 1. Include all members of the TAND consortium in the consensus panel

The TAND consortium included a group of 24 individuals from all World Health Organization (WHO) regions but one (Eastern Mediterranean), across multiple professional groups including psychiatry, psychology, pediatric neurology, nephrology, speech and language therapy, education, special education, intellectual disability medicine, engineering, and neuroscience [9] (www.tandconsortium.org). The consortium also included “family representatives” of individuals with TSC or family members of people with TSC. Many of the family representatives had “lived expertise” as well as other professional expertise, such as in education or health services. All consortium members participated in the process. Here, we will refer to this group as the “consensus panel.”

### Step 2. Creation of cluster groups

Previous research identified seven natural TAND clusters [20]. We therefore divided consortium members into cluster groups for each natural cluster based on areas of expertise and interest. In addition, we created a cluster group for the psychosocial level, given that psychosocial difficulties (the psychological impact of living with TSC and TAND) were not included in the generation of TAND clusters [18–21]. Each of the eight cluster groups had a lead and a co-lead, plus one or more additional members (for details see [9]). Every cluster, with the exception of the eat/sleep cluster, included at least one family representative. Table 1 shows the eight clusters, the consortium members in each cluster group, and the items from the TAND-L Checklist included in the cluster.

**Table 1:** Cluster groups in the study

Cluster	Members	TAND Checklist items
Autism-like	Nola Chambers (lead), Jamie Capal (co-lead), Eva Schoeters, Sebastián Cukier, Shoba Srivastava	• Absent or delayed onset of language to communicate • Repeating words or phrases over and over again • Poor eye contact • Difficulties getting on with other people of similar age • Repetitive behaviors, such as doing the same thing over and over again • Very rigid or inflexible about how to do things or not liking change in routines
Dysregulated behavior	Tanjala Gipson (lead), Peter Davis (co-lead), Agnies van Eeghen	• Aggressive outbursts • Temper tantrums • Self-injury, such as hitting self, biting self, scratching self
Eat/sleep	Stacey Bissell (lead), Katie Smith (co-lead), Peter Davis	• Difficulties with eating, such as eating too much, too little, unusual things • Sleep difficulties, such as falling asleep or waking
Mood/anxiety	Agnies van Eeghen (lead), Jamie Capal (co-lead), Megumi Takei, Robert Waltereit	• Anxiety • Depressed mood • Extreme shyness • Mood swings
Neuropsychological	Anna Byars (lead), Jennifer Flinn (co-lead)	• Memory, such as remembering things that have happened • Attention, such as concentrating well, not getting distracted • Dual-tasking/Multi-tasking, such as doing 2 tasks at the same time • Visuo-spatial tasks, such as solving puzzles or using building blocks • Executive skills, such as planning, organizing, flexible thinking • Getting disoriented, such as not knowing the date or where you are
Overactive/impulsive	Robert Waltereit (lead), Stacey Bissell (co-lead), Katie Smith, Megumi Takei	• Overactivity/hyperactivity, such as being constantly on the go • Restlessness or fidgetiness, such as wriggling or squirming • Impulsivity, such as butting in, not waiting turn
Scholastic	Jennifer Flinn (lead), Peter Davis (co-lead), Shoba Srivastava	• Reading • Writing • Spelling • Mathematics
Wraparound psychosocial	Stephanie Vanclooster (lead)*; Sebastián Cukier (lead)**, Chris Kingswood (co-lead), Eva Schoeters, Katie Smith	• Both for individuals with TSC and their caregivers: • Low self-esteem • Very high levels of stress in the family • Very high levels of stress in relationship with siblings • Very high levels of parent–child relationship difficulties • Very high levels of parent-to-parent/partner relationship difficulties • Very high levels of stress leading to difficulty for family to connect with others in the community • Very high levels of stress leading to difficulty to progress in career

^*^Lead from 2019 to 2021; **Lead from 2021

### Step 3. Review of the literature

The TAND literature was reviewed in two ways. First, a comprehensive scoping review of all TAND research ever published in the peer-reviewed literature was conducted by the consortium [27]. The purpose of the scoping review was to provide an unbiased review of the TSC-specific evidence (or lack thereof). Secondly, cluster teams each performed a targeted review that focused on literature within and outside TSC that was felt to be relevant and important by the cluster team. These reviews were therefore not highly systematic, but focused on systematic reviews, meta-analyses, and widely accepted clinical guidelines (e.g., NICE guidelines, APA practice parameters). Publications were not only focused on English-speaking healthcare settings, given the international nature of the consortium.

### Step 4. Generation of cluster chapters, summary statements, and recommendations

Cluster groups used all available TSC literature and relevant non-TSC literature to draft a “cluster chapter,” as well as summary statements and cluster-based recommendations based on the literature review and their expert opinion. Each cluster chapter was reviewed by two reviewers from other clusters, and improvements were incorporated into cluster chapters, summary statements, and draft recommendations.

### Step 5. Presentation and review of summary statements and recommendations per cluster

All cluster chapters, summary statements, and recommendations were made available to the consensus panel. Over a period of 3 months, each cluster group presented their summary statements and recommendations in a series of online meetings. Consensus panel members questioned cluster teams to seek clarification, items were discussed, and text was revised based on discussions. Overall, this process allowed for an iterative and international review of relevant literature given the global goal of our recommendations.

### Step 6. Electronic voting and prioritization of summary statements and recommendations

All members of the consensus panel were provided with an online survey which included all summary statements and recommendations—members were asked to vote “strongly agree,” “agree,” “disagree,” or “strongly disagree” on each. They were also asked to provide suggestions if they felt modifications to a statement or recommendation were required and to prioritize their top three statements and recommendations per cluster. Data were collated by the TANDem Action Group in preparation for a 3-day online consensus conference.

### Step 7. Consensus conference

At the online consensus conference with the consensus panel, data from votes (as outlined in step 6) were presented, and items where consensus could not be reached even after modification and discussion were excluded.

### Step 8. Identification of core principles

It became clear during the evidence review and consensus-building process that a number of core principles were emerging across all clusters. The consortium conference therefore also included the generation of potential core principles from all cluster-specific recommendations.

### Step 9. Electronic voting and prioritization of core principles

All potential core principles were collated and consortium members were asked to vote in an online survey whether they “strongly agree,” “agree,” “disagree,” or “strongly disagree” with the proposed principles. From 18 potential principles, a final list of ten core principles was generated with 100% consensus.

### Step 10. Generation of the final set of evidence-informed consensus recommendations for the identification and treatment of TAND

The process outlined above led to a final set of recommendations including 10 core principles and eight sets of cluster-specific recommendations. The psychosocial cluster was conceptualized as a “wraparound” cluster (encompassing and relevant to all natural clusters). All consensus panel members reviewed and approved all recommendations.

## Results

**Fig. 2:**
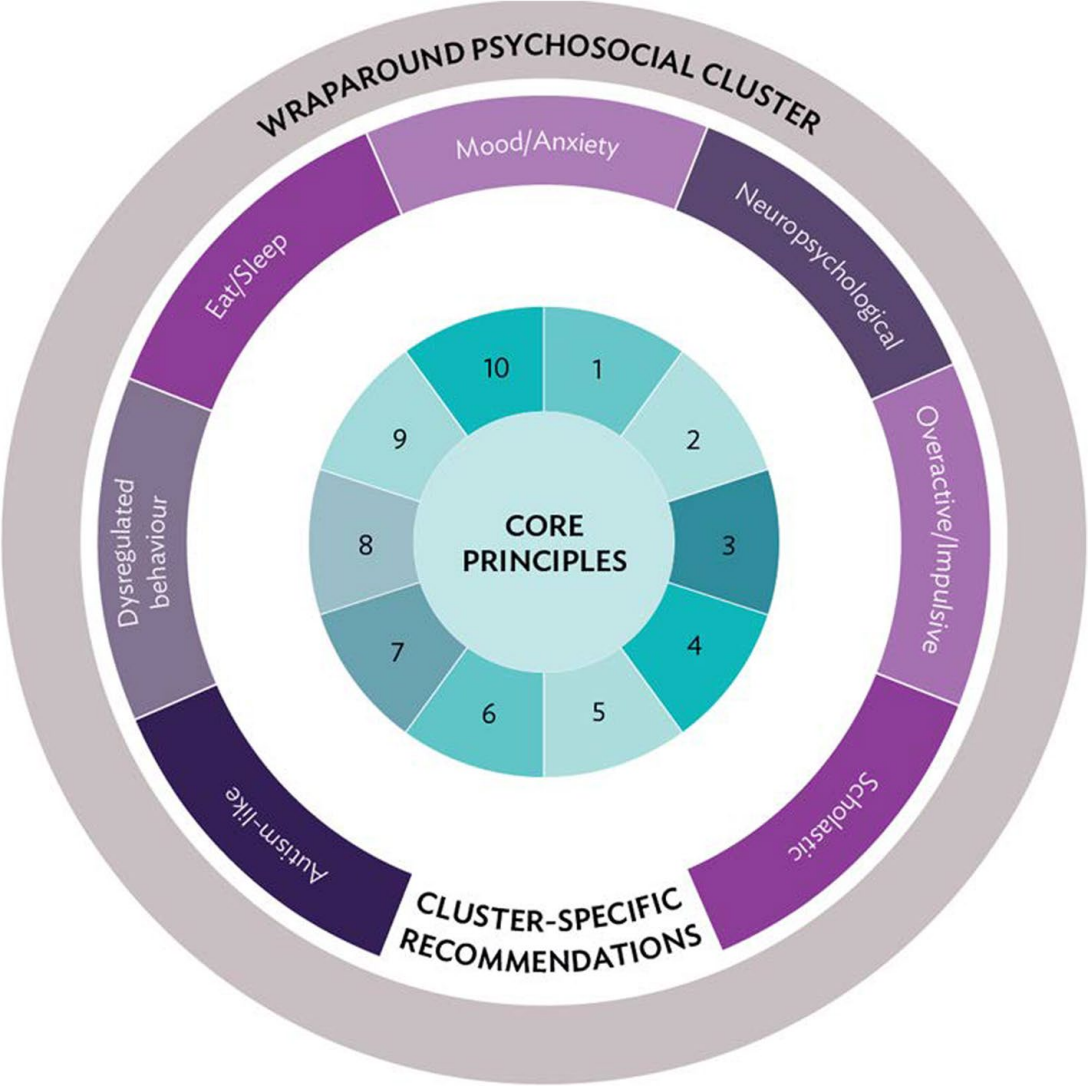
Visual summary of the consensus recommendations for TAND. Recommendations include ten core principles (outlined in Table 2), seven sets of cluster-specific recommendations (outlined in Tables 3, 4, 5, 6, 7, 8 and 9), and wraparound psychosocial recommendations (outlined in Table 10)

Figure 2 shows a conceptual representation of the evidence-informed consensus clinical recommendations. At the heart of the recommendations are ten core principles to be used by clinicians and families as an overall guide to the identification and treatment of TAND. This is surrounded by cluster-specific recommendations for each of the seven natural TAND clusters. Around all these recommendations, the wraparound psychosocial cluster recommendations were placed to show how these “wrap around” all the core principles and cluster recommendations.

### Core principles for the identification and treatment of TAND

The ten core principles for identification and treatment of TAND are shown in Table 2 and are proposed as a framework to approach any individual with TSC, regardless of their age, sex, genotype (e.g., *TSC1* or *TSC2*), or TAND profile. It recognizes that everyone with TSC is at risk of TAND manifestations (#1) and that it is therefore important to perform lifelong monitoring for the emergence of TAND difficulties (#2) with a minimum of annual screening (#3). The core principles recommend early identification and early intervention (#4) rather than to use a “watch-and-wait” strategy. The consensus panel proposes a cluster-based profiling and identification of needs, but acknowledges that clusters cluster together and that identification of needs in one cluster should also alert caregivers and clinical teams to explore other clusters (#5). In the context of a multi-system condition, core principle #6 points to the importance of the relationship between physical health manifestations and TAND manifestations. Core principle #7 underlines the importance of working with caregivers as lived experts in TSC. Recognizing the very limited traditional “evidence base” in TAND, caregivers, and family communities have valuable contributions to make both in identification and in intervention for TAND. Principle #8 serves as a reminder that TAND difficulties require a “whole-system” understanding to guide an intervention plan. This means that biological, psychological, and social (“bio-psycho-social”) aspects should be considered to understand the needs and to provide interventions and support. By implication, this process requires contributions from many professional groups and disciplines and is not just about finding a “medication” to solve a problem. In spite of the limited evidence base from the scientific literature specifically on TAND, principle #9 recommends the need to be informed by whatever relevant evidence may exist and to guard against approaches known to have evidence of harm. The final principle (#10) is a reminder that the goal of “intervention” and support to individuals with TSC and their caregivers is not merely to reduce symptoms or difficulties, but to help everyone living with TSC achieve an optimal quality of life as individuals and as families and to facilitate their active participation in all aspects of society throughout their journey with TSC and TAND.

**Table 2:** The ten core principles for the identification and treatment of TSC-associated neuropsychiatric disorders (TAND)

**1. Everyone with TSC is at risk of TAND.** Most people with TSC will have some TAND manifestations at some point in their lives. There are risk markers that increase the likelihood of TAND manifestations (such as the presence of intellectual disability, seizure disorders, a pathogenic variant in *TSC2* vs *TSC1*). However, even without these risk markers, people can have TAND manifestations. Every person with TSC should therefore be considered as being at risk for TAND
**2. Everyone with TSC needs lifelong monitoring for the emergence of TAND.** Given that everyone is at risk of some TAND manifestations and given that TAND may present at various times throughout the lifespan, all people with TSC need lifelong monitoring for the emergence of TAND. Monitoring means screening at least annually to look for possible TAND manifestations in a systematic way. Tools like the TAND-L or TAND-SQ Checklists are well suited for regular screening and can be used by any professional who is supporting an individual who lives with TSC (TAND-L) or by families themselves (TAND-SQ)
**3. Screen at least annually and follow up with appropriate action.** Whenever screening picks up any concerns, this should be followed by appropriate action. Any concern about any TAND cluster difficulties should lead to an appropriate next step. Some of the next steps may be things to seek out (e.g., referral to a healthcare professional for diagnostic work-up and appropriate intervention based on the outcome of that evaluation). Other next steps may be things caregivers can do themselves (e.g., self-help tools or home-based interventions). All diagnoses and interventions should be provided by suitably qualified professionals. However, around the globe there are differences in which professional group does what. Screening should then be repeated at least once per year to make sure emerging difficulties are identified as soon as possible
**4. The goal is early identification and early intervention.** Given that all people with TSC are at risk of TAND, the goal is to identify TAND difficulties as early as possible and then to provide intervention as early as possible. Caregivers and clinicians are encouraged not to ‘watch and wait’ to see if things get better, but to act swiftly if there is concern
**5. TAND clusters cluster together.** It is very common for people with TSC to have difficulties in multiple clusters. If difficulties are identified in one TAND cluster, look for difficulties in other clusters. Co-occurring difficulties in TSC is the rule rather than the exception. Where cluster difficulties co-occur, it may be important to think how to prioritize and coordinate interventions
**6. Always consider the impact of physical health problems and medications for physical health problems on TAND.** Most people with TSC will have physical health difficulties such as skin, brain, kidney or other organ system involvement. These may play a very important role in relation to TAND manifestations. For instance, they may be the direct causes of a TAND difficulty (e.g., a growing Subependymal Giant Cell Astrocytoma, SEGA), or the medications used to treat the physical condition may cause TAND (e.g., antiseizure medications). Whenever anyone has TAND difficulties, consider the role of physical health problems and medications first. This is particularly important when sudden and unexpected change is seen in the TAND profile of a person with TSC
**7. Work with families and caregivers as lived experts in TSC and TAND.** Caregivers and people with TSC live with their difficulties every day. They are therefore the primary agents for the identification of TAND difficulties. They are also the most important partners in interventions for TAND. It is therefore of fundamental importance to strengthen and support families and to maintain a healthy partnership with family caregivers and individuals with TSC, recognizing their “lived expertise” with TSC and TAND. Some interventions should be provided by professionally qualified individuals. However, there are many evidence-based interventions that can be led by caregivers and families
**8. Generate a “bio-psycho-social” “whole-system” plan for intervention.** All TAND intervention plans should consider all the potential contributing factors such as biological (e.g., role of physical health, TSC medications, co-occurring diagnoses), psychological (e.g., family stress, life events, personality, and parenting styles), and social factors (e.g., need for financial support, other social, or environmental factors). Intervention is therefore a broad concept and is not just about medication. By having a “whole-system” approach, caregivers and TSC teams will think how to integrate healthcare, education, social wellbeing, and community participation into the intervention for the individual and family who live with TSC
**9. Be evidence-based and evidence-informed.** Even though there are many gaps in the TSC evidence base, specifically for the identification and treatment of TAND, all professionals should use evidence-based strategies as recommended in the general population and make recommendations in an evidence-informed manner, rather than use or recommend strategies or interventions that have no evidence-base or that may be harmful. In discussions with families, all professionals should be clear when advice is based on established evidence and when not
**10. Strive for optimal functional outcomes and quality of life throughout the journey with TSC and TAND.** The purpose of diagnosis and treatment should not simply be symptom control or removal of disorder/disease, but to achieve the optimal functional outcomes for individuals and families—this should include activities and participation (including education, occupation, and leisure), social inclusion (for the individual, caregivers, and family) and optimal quality of life (for the individual, caregivers, and family). Across the journey with TSC and TAND, different outcomes may become priorities, and different elements will ensure a good quality of life. These require review and re-focusing, particularly at key transitional timepoints, for instance moving from pre-school to school, moving out of formal education, and so on

### Cluster-specific recommendations

Consensus recommendations for the seven natural TAND clusters and the wraparound psychososocial cluster are presented in the text below and in Tables 3, 4, 5, 6, 7, 8, 9 and 10.

### Autism-like cluster

The autism-like cluster recommendations are shown in Table 3. TSC is associated with very high rates of social-communication difficulties and a significant proportion of individuals with TSC meet the criteria for autism as defined in the DSM-5 or ICD-11 [16, 28–31]. However, these difficulties are often identified or diagnosed late, and many children and families miss out on opportunities to access some of the growing number of evidence-based interventions and support programs developed specifically for autism [4]. Similarly, adults with TSC who have social-communication difficulties or autism rarely receive interventional support.

**Table 3:** Autism-like cluster recommendations

**AU1. Monitor all individuals with TSC for manifestations in the autism-like cluster.** The rates of autism-like cluster manifestations are high in individuals with TSC across age, sex, and developmental level with rates ranging from 41 to 69%. For this reason, all individuals with TSC (children and adults) should be monitored for manifestations in the autism-like cluster, including social communication and language difficulties, and the presence of repetitive and restricted behaviors
**AU2. Monitoring for autism-like manifestations should start in early infancy and continue through adulthood.** In people with TSC, manifestations in the autism-like cluster may emerge very early in development, or manifest throughout the developmental period. These difficulties may continue into adulthood and may have a negative impact on other aspects of functioning. For this reason, developmental monitoring and surveillance for autism-like manifestations should begin very early in all children with TSC to ensure early detection and access to early intervention. Surveillance should continue into adult life to provide supports for these difficulties
**AU3. All individuals with autism-like cluster manifestations should be referred for a diagnostic assessment for communication disorders and autism/autism spectrum disorder (ASD).** The rates of formal clinical diagnoses of autism are approximately 40–50% in children and adolescents with TSC, which is substantially higher than in the general population (1–2%). For this reason, all individuals who present with some autism-like cluster manifestations should receive a comprehensive diagnostic work-up for communication disorders and autism, as well as for all co-occurring conditions often associated with autism
**AU4. The literature and clinical guidelines developed for children and adults with autism in the general population may be relevant and applicable to those with autism in TSC.** A small number of studies, mostly in children, suggest that autism in individuals with TSC looks very similar to autism in those without TSC (referred to as “idiopathic” or “non-syndromic” autism). For this reason, the clinical and interventional guidelines for autism may be relevant for the identification and intervention of autism in TSC
**AU5. Interventions using Naturalistic Developmental Behavioral Interventions (NDBIs) may improve social communication and behavioral outcomes in young children with autism in TSC.** There are numerous evidence-based interventions that target and improve the core features of autism in the non-TSC literature. NDBIs represent the group of non-pharmacological interventions that have the strongest evidence base to date. NDBIs can be delivered by clinicians or by caregivers in their natural environments. These may therefore be of clinical benefit also to children with autism in TSC and their caregivers
**AU6. Adults with TSC and autism-like cluster manifestations may benefit from recognized autism interventions, particularly social skills interventions.** There is some evidence of the effectiveness of interventions for adults with autism in the general population to improve spoken language and social skills. Even though there is no TSC-specific evidence to date, such interventions may be helpful to adults with TSC and autism-like cluster manifestations
**AU7. All children and adults with TSC and autism-like cluster manifestations or autism diagnoses should be monitored for common co-occurring psychiatric, developmental, and physical health disorders and appropriate treatment should be provided.** Many individuals with autism without TSC present with a range of co-occurring neurodevelopmental, mental health, and physical health disorders that may negatively impact their developmental progress and behavior. These co-occurring disorders are treatable yet frequently go undetected. The same principle applies to autism in TSC. For this reason, all children and adults with TSC and autism-like cluster manifestations should be monitored for these common co-occurring conditions. Identification should lead to prompt and evidence-based treatments
**AU8. The autism-like cluster and autism in TSC is an important area for future research.** Even though the autism-like cluster and autism in TSC have received more research relative to most other TAND clusters, the evidence base, particularly for interventions, remains very limited. There are almost no studies documenting the effectiveness of non-pharmacological or early behavioral interventions for young children with TSC showing autism-like cluster manifestations or with a diagnosis of autism. Given the high rate of autism in TSC, this is therefore an important area in need of future research. In the meantime, we recommend the evidence and consensus clinical guidelines for autism in the general population for guidance

For these reasons, the consensus panel recommended lifelong monitoring of all individuals for manifestations in the autism-like cluster (AU1), from early in infancy and throughout adulthood (AU2). All individuals who show difficulties with autism-like cluster manifestations should be referred for a formal clinical evaluation for communication disorders and autism/autism spectrum disorder (AU3). In the absence of TSC-specific evidence, the consensus panel recommended that the autism literature on children and adults in the general population (i.e., for those with autism without TSC) may be relevant to the TSC community (AU4). For this reason, young children with autism and TSC may benefit from naturalistic developmental behavioral interventions (NDBI) [32, 33], the group of autism interventions with the strongest evidence base at present (AU5). Similarly, adults with TSC and autism-like cluster manifestations may benefit from autism interventions such as social skills training (AU6) [33]. Recognizing the very high rates of co-occurring physical health, neurodevelopmental, and mental health problems in autism in the general population [33], as well as the common co-occurrence of other TAND clusters with the autism-like cluster [18], the panel recommended lifelong monitoring for the presence of co-occurring conditions, followed by appropriate evidence-informed treatments (AU7). Even though autism and the autism-like cluster have been the most extensively examined in TSC research [27], much further research is recommended, particularly in relation to non-pharmacological interventions for difficulties in this cluster (AU8).

### Dysregulated behavior cluster

The dysregulated behavior cluster recommendations are shown in Table 4. Difficulties with aggression, temper tantrums, and/or self-injurious behaviors represent some of the greatest concerns and burdens to families who live with TSC [29]. These behaviors are therefore a common reason for referral to specialist services. However, there may be many different reasons for or “pathways” to dysregulated behaviors in TSC. For example, they may be driven by communication difficulties, impulsivity, anxiety, sensory sensitivities, demand avoidance, cognitive inflexibility, trauma, and/or pain [34–37]. Dysregulated behaviors may also emerge as a result of the physical manifestations of TSC, such as growing SEGA, seizures, or as an adverse effect of medications. For this reason, there is no single intervention approach to this cluster of difficulties, and, equally, no single or simple medication that should be used to “manage” these behaviors. There are also no behavioral treatment studies for dysregulated behavior specifically in people with TSC. However, there is moderate support for real interventions for individuals with intellectual disabilities without TSC, which should inform practice [38]. Non-pharmacological/behavioral interventions may include speech/language work to support communication difficulties, cognitive-behavioral work to support anxiety behaviors and cognitive inflexibility, a range of sensory strategies to support the sensory sensitivities that may trigger dysregulated behaviors, and a range of environmental strategies such as visual schedules to increase predictability and support transitions during daily activities [34–38].

**Table 4:** Dysregulated behavior cluster recommendations

**DB1. Dysregulated behaviors are common in TSC, have a major impact on the family, and require a systematic approach.** Dysregulated behaviors, including aggression, temper tantrums, and self-injury, are common in individuals with TSC and can have a major impact on the family. Most of the time, dysregulated behaviors have a “meaning” or “function” and are not due to the individual being intentionally difficult. Many behaviors have specific triggers, purposes, or are reinforced by the responses of others to the behavior. Caregivers should therefore be supported to receive a systematic and comprehensive evaluation to understand the dysregulated behaviors
**DB2. Understand the meaning/function of dysregulated behaviors to guide intervention.** There may be many different reasons why individuals with TSC may present with dysregulated behaviors. These include (but are not limited to) difficulties with communication, overactive and impulsive behaviors, mood and anxiety problems, sensory sensitivities, avoidance of demand, cognitive inflexibility, or pain. The intervention plan for each of these reasons may be very different. For this reason, caregivers should be supported to complete a functional behavioral assessment to identify antecedents and consequences of specific behaviors as a way of understanding the reason, function, or meaning of a specific dysregulated behavior
**DB3. Understand the intellectual and neuropsychological profile to inform the meaning/function of dysregulated behaviors and to guide intervention.** Ensure a good understanding of the intellectual ability and neuropsychological profile of an individual with dysregulated behaviors as these may inform the reason, function, or meaning of such behaviors. Where necessary, perform formal assessments to evaluate current intellectual, language/communication, attention, executive, memory, and visuospatial skills so that goals and support can be adapted appropriately for each individual. Provide accommodations and resources to optimize communication (e.g., hand signs, picture cards, devices/apps) and other skills as required
**DB4. Perform urgent physical work-up for sudden onset of dysregulated behaviors and/or rapid change in behaviors.** Sudden onset or change in behaviors may indicate an underlying medical problem, including (but not limited to) seizures, subependymal giant cell astrocytoma (SEGA), physical illnesses, or adverse effects of medications. Look out for new or alarming physical or neurological symptoms such as lethargy, confusion, vomiting, or physical pain, and perform urgent medical/neurological workup to identify potential physical causes of dysregulated behaviors
**DB5. Use non-pharmacological intervention strategies as first-line treatment for dysregulated behaviors.** Once a functional analysis of behaviors has been performed and underlying medical causes for behaviors have been ruled out, non-pharmacological strategies should be implemented as a first-line treatment. These are determined by the meaning or function of the behavior, and typically include communication, behavioral, developmental, and environmental strategies. These non-pharmacological strategies are often more effective than medication and should always be part of treatment plans
**DB6. Medications should only be used for dysregulated behaviors after a careful systematic evaluation and always alongside a non-pharmacological intervention plan.** There are no medication strategies that will improve dysregulated behaviors in isolation. Where medications are considered, they should always be integrated in a broader management plan that includes non-pharmacological approaches. Medications should always be prescribed within the marketing authorization of the drug, for as short a period as possible, and with clear consent from the individuals with TSC or caregivers
**DB7. Research is required to generate a TSC-specific evidence base for non-pharmacological interventions of dysregulated behaviors.** There is a gap in research for TSC-specific behavioral and other non-pharmacological interventions. In the interim, we recommend the use of guidelines for the management of dysregulated behaviors in the general population

For these reasons, the consensus panel stated that dysregulated behaviors are common, have a major impact on families, and should be investigated in a systematic way (DB1). Given the many possible underlying meanings or functions of behaviors, a careful and systematic functional analysis of behavior should be conducted to generate an understanding of the problem behavior (DB2) [39]. Another important step towards a good understanding is to conduct an evaluation of the intellectual and neuropsychological profile of the individual with TSC (DB3). To ensure rapid identification of any underlying biological cause of dysregulated behaviors, urgent physical examination is recommended for sudden onset and/or rapidly changing or unexpected dysregulated behaviors (DB4). Once the evaluation has been completed, biological causes identified and treated, and a good understanding of the pathways to the dysregulated behavior has been identified, non-pharmacological strategies are recommended as first-line treatment (DB5). Medications should only be used for dysregulated behaviors after a careful and systematic evaluation and always alongside a non-pharmacological intervention plan (DB6). The consensus panel also recognized the need to generate an evidence base particularly for non-pharmacological interventions for dysregulated behaviors in individuals with TSC (DB7).

### Eat/sleep cluster

The eat/sleep cluster recommendations are shown in Table 5 with separate recommendations for eating- and sleep-related difficulties. Eating difficulties in TSC are a highly under-researched domain, but the consensus panel recognized that they do occur and may be associated with a range of TAND and/or physical manifestations (ES1). Where eating difficulties are reported, a comprehensive evaluation should be performed to consider the range of potential contributors (e.g., picky eating, autism-related restricted eating, mouth ulcers or anorexia associated with medications, physical ill-health, or pain) (ES2). Recognizing the wide range of developmental, intellectual, and communication levels in TSC, intervention plans for eating should be adapted to the individual needs and profile of each person with TSC. The consensus panel stated that there are no dietary supplements or restricted/special diets with an evidence base in TSC to improve any TAND manifestations (ES3). The ketogenic diet is a well-known approach used for refractory seizures in TSC, but the evidence that it has a direct impact on TAND is mixed [40].

**Table 5:** Eat/sleep cluster recommendations

**ES1. Eating difficulties do occur in TSC and may be associated with a range of TAND and physical health manifestations.** There is limited literature on eating-related difficulties and disorders in TSC, but difficulties with eating do present in individuals with TSC. Eating difficulties in TSC may be similar to those seen in typically developing children (e.g., picky eating), be associated with TAND-related manifestations (e.g., autism-related restricted eating, or mood and anxiety-related over/under-eating), or be associated with physical health in TSC (e.g., mouth ulcers or other adverse effects of medications, physical ill health, pain). Efforts should therefore be made to monitor eating and changes in eating via self-report or informant-report measures on a regular basis
**ES2. Eating difficulties in TSC require a comprehensive workup and intervention plan in the context of the range of typical TAND and physical health-related associations.** Given that eating difficulties and eating disorders in TSC may be associated with a range of factors, it is important to generate a comprehensive evaluation of the difficulties to inform an appropriate intervention plan. Interventions may include non-pharmacological and pharmacological strategies, depending on the causes of the eating difficulties. The intervention plan should be adapted to the intellectual and communication profile of the individual
**ES3. There is no scientific evidence to recommend over-the-counter supplements or restricted diets for TAND.** In spite of the interest in the popular literature, there is no scientific evidence base to recommend specific supplements or any specific diets (e.g., gluten-free/casein-free) to improve TAND manifestations. The ketogenic diet is used as an intervention for refractory seizures in TSC, but not as a dietary intervention for TAND
**ES4. Sleep difficulties are common in children and adults with TSC across age, sex, and genotype and should be monitored on a regular basis.** Sleep difficulties are common in children and adults in the general population but are more pronounced in TSC. Efforts should therefore be made to monitor sleep and changes in sleep via self-report or caregiver-report measures on a regular basis
**ES5. Sleep difficulties may be a “cause” and/or a “consequence” of TAND and other neurological manifestations and should be evaluated with this in mind.** Sleep difficulties may be a “cause” of some TAND and other neurological manifestations (e.g., leading to dysregulated or overactive and impulsive behaviors, poor scholastic performance, or seizures). Sleep difficulties could also be a “consequence” of TAND and neurological manifestations (e.g., autism-related rigid sleep routines, mood and anxiety-related insomnia, waking due to nocturnal seizures, or adverse effects of medications). This “bidirectional” association should therefore be considered during evaluation and intervention planning
**ES6. Healthcare providers should first investigate and treat the biological and psychiatric causes of sleep difficulties before proceeding to non-pharmacological/pharmacological treatments of the sleep.** Healthcare providers and caregivers should first evaluate and treat the biological and psychiatric “causes” of sleep problems in TSC, which may include seizures, pain, adverse effects of prescribed medications, or mood and anxiety disorders, before introducing behavioral/non-pharmacological and/or pharmacological treatments for sleep difficulties
**ES7. Non-pharmacological strategies should be used before pharmacological strategies to manage sleep difficulties.** Once other causes of sleep problems have been identified and treated, or excluded, non-pharmacological strategies should be implemented as the first-line approach. These may include sleep education, behavioral/environmental modifications, and sleep hygiene practices from early in life. For example, consistent bedtime routines, conducive sleeping environments, and limiting access to technology before bedtime should be implemented before the use of pharmacological interventions is considered
**ES8. There is a need for targeted research on eating and sleep difficulties in TSC.** Despite the significant impact of eating and sleep difficulties on individuals and families with TSC, there is a very limited research base. The prevalence of clinical eating disorders and sleep disorders in TSC is unknown. Diagnoses and treatment recommendations from the general literature should be used and adapted in the context of co-occurring physical health and TAND manifestations seen in TSC

Sleep difficulties are very common across all ages in TSC [4, 41], but there is a complex “bidirectional” association between sleep and other manifestations. For example, sleep difficulties may contribute to neuropsychological difficulties (e.g., in memory or attention), dysregulated behaviors (e.g., increased aggression or temper tantrums), mood problems, or seizures (acting to reduce seizure thresholds or acting as trigger events). Conversely, sleep difficulties may result from other TAND manifestations (e.g., autism-related rigid sleep routines, mood/anxiety-driven insomnia, or early morning wakening), neurological manifestations (e.g., waking from a nocturnal seizure), or result from adverse effects of medications. Sleep difficulties may also be maintained by a behavioral model of reinforcement, such as access to electronic devices or caregiver contact upon waking, or an inadvertent mutual reinforcement cycle including caregivers (e.g., co-sleeping in the caregivers’ bed to help them settle). Pathways to sleep difficulties are complex, and assessment strategies therefore need to be set up to understand each individual’s pathways to their sleep difficulties, in order to ensure appropriate management.

For these reasons, the consensus panel emphasized that sleep difficulties should be monitored regularly regardless of the age, sex, and genotype (ES4) of individuals and that sleep difficulties may be a “cause” and/or a “consequence” of TAND or other neurological manifestations (ES5). Clinicians and caregivers should therefore perform careful examinations to first identify and treat biological contributors to sleep, such as underlying health conditions and behavioral markers of pain (ES6). Next, psychiatric contributors to sleep should be examined and treated (e.g., early morning wakening as part of a depressive disorder or disturbed sleep in the context of an anxiety disorder). Only once these have been identified and treated (or excluded), should other sleep management strategies be explored. Non-pharmacological strategies should always be used first (e.g., sleep education, sleep hygiene) before pharmacological strategies (e.g., melatonin or similar medications) are considered (ES7). Given the very limited evidence base in this cluster, the consensus panel recommended targeted research on eating and sleeping difficulties in TSC (ES8).

### Mood/anxiety cluster

The mood/anxiety cluster consensus recommendations are shown in Table 6. The rates of mood and anxiety symptoms and disorders are very high in TSC, often arising in adolescence or adulthood. Difficulties in this cluster are often identified late or not at all. In those with developmental or intellectual disabilities, the identification of mood and anxiety difficulties may be even more difficult. Even though there is no evidence base within TSC for the treatment of depressive and anxiety disorders, there is an encouraging evidence base in the general population that indicates the use and effectiveness of non-pharmacological and pharmacological strategies.

**Table 6:** Mood/anxiety cluster recommendations

**MA1. Mood and anxiety symptoms should be monitored in all children and adults with TSC to ensure early detection and treatment when necessary.** The rates of mood and anxiety symptoms are very high in individuals with TSC (up to 56% overall). Mood and anxiety symptoms can emerge very early in development but are more commonly seen in adolescents and adults with TSC. For this reason, regular assessment of mood and anxiety symptoms should be performed to identify emerging difficulties. It may be appropriate to refer to a psychiatrist for formal assessment and diagnosis of a mood or anxiety disorder if symptoms are persistent, severe, and impairing functioning in the individual
**MA2. Mood and anxiety disorders are highly underdiagnosed and under-treated in TSC, particularly in individuals with intellectual and other neurodevelopmental disabilities.** Mood and anxiety disorders are often under-diagnosed in individuals with TSC, particularly in those with intellectual disability as it may be difficult for them to communicate such difficulties appropriately. These difficulties may present differently in individuals with intellectual and developmental disabilities (e.g., as a change in typical behaviors, withdrawal from activities, or reduced enjoyment of previously motivating activities). This may result in under-identification and under-treatment of these disorders. It is therefore important for caregivers and healthcare providers to be vigilant in inquiring about mood and anxiety symptoms in all individuals with TSC to ensure early identification of such concerns
**MA3. Mood and anxiety symptoms may present as manifestations of physical health disorders and/or as adverse effects of prescribed medications.** Individuals with TSC often have epilepsy and other physical health disorders. The role of seizures, other health conditions, and prescribed medications should therefore be considered as possible contributors to the mood/anxiety profile of an individual during clinical evaluation
**MA4. Mood and anxiety should be treated using evidence-based approaches recommended in the general population.** Even though there is no specific evidence-base for interventions for mood and anxiety disorders in TSC, there is a strong evidence-base for treatments of these manifestations in the general population. For treatment of mild to moderate mood and anxiety disorders in individuals with TSC, non-pharmacological approaches are recommended, such as physical activity and cognitive behavioral therapy. Nonverbal therapies (psychomotor therapy, creative therapy, mindfulness) and the adjustment of contextual factors may also be part of first-line treatment. When these non-pharmacological approaches are insufficient, or in the case of severe mood and anxiety disorders, these strategies should be combined with an evidence-based pharmacological treatment, such as selective serotonin reuptake inhibitors (SSRIs), or serotonin and noradrenalin reuptake inhibitors (SNRIs). Diagnosis and treatment of mood and anxiety disorders should always be done in collaboration with a qualified mental health professional
**MA5. Mood and anxiety disorders should be managed using a personalized approach.** The individual’s profile of needs will be influenced by many factors, including co-occurring TAND and physical health problems, age and developmental level, personal, family, and psychosocial factors. All these should be integrated to plan a personalized approach to intervention
**MA6. Further research is needed to generate an evidence base for identification and treatment of mood and anxiety difficulties and disorders in TSC.** In spite of high rates of mood and anxiety difficulties and disorders in TSC, the research evidence base remains very limited. For example, there are no TSC-specific data to inform targeted pharmacological or non-pharmacological interventions for mood and anxiety disorders. Further research is therefore clearly needed

For these reasons, the consensus panel recommended that all children and adults should be monitored for the emergence of mood and anxiety symptoms to ensure early detection and treatment (MA1). Particular efforts should be made to look for mood and anxiety symptoms in those with intellectual and other neurodevelopmental disabilities where manifestations of depressed mood or anxiety may be different (e.g., withdrawal from social interaction, loss of interest in previously enjoyed activities, anorexia, or increased dysregulated behaviors) (MA2). Mood and anxiety symptoms may be the consequence of underlying physical health problems or their treatments (e.g., seizures and anti-seizure medications, renal failure, or chronic pain), and these may require specific management (MA3). Where mood and anxiety symptoms are identified, an appropriate diagnostic evaluation should be performed, and evidence-based non-pharmacological and pharmacological interventions as recommended in the general population should be used to treat mood and anxiety disorders (MA4). Given the heterogeneity in the individual physical health and TAND profiles of individuals with TSC, a personalized approach to management is recommended (MA5). In spite of the high rates of mood and anxiety disorders in TSC, the research evidence base is very limited, and further research, particularly interventional research, was recommended (MA6).

### Neuropsychological cluster

The neuropsychological cluster consensus clinical recommendations are shown in Table 7. About half of the individuals with TSC have normal-range intellectual ability (with IQ > 80) and 40–50% have intellectual disabilities. However, the individual profiles of strengths and weaknesses are highly variable between individuals and are often very uneven within individuals regardless of their “overall” intellectual ability [4, 15, 29, 42]. Intellectual ability is a very strong correlate of many TAND manifestations, and uneven intellectual profiles can be associated with many functional impairments. Even in people with above-average and high intellectual abilities, the rates of specific neuropsychological deficits (e.g., in attentional, memory, or executive skills) are very high and can be associated with significant challenges in daily life (e.g., in school, relationships, or the workplace) [43, 44]. This is even more likely to be the case for those with TSC known to have neurodevelopmental disorders such as autism, attention deficit hyperactivity disorder (ADHD), or learning disorders. Understanding the neuropsychological profile of an individual with TSC can help to understand current difficulties and to predict future ones. Performing these evaluations in preparation for transitions in school, in preparation for post-secondary training or for the workplace, and implementing neuropsychological intervention plans, can be of significant value.

**Table 7:** Neuropsychological cluster recommendations

**NP1. All individuals with TSC should receive an assessment of their intellectual ability to identify their profile of strengths and weaknesses.** The majority of individuals with TSC have normal-range intellectual ability (IQ > 80), and 40–50% have intellectual disability (IQ < 70 and functional impairment in adaptive behavioral skills). Regardless of their level of intellectual ability, the majority of people with TSC have a very uneven profile of intellectual strengths and weaknesses. Intellectual ability is a very strong predictor of many TAND manifestations, and uneven intellectual profiles can be associated with many difficulties in daily life functioning, such as in school or work. For this reason, all individuals with TSC should receive a comprehensive assessment of their intellectual ability at the time of diagnosis using age- and developmentally appropriate measures. Evaluations should be repeated as clinically indicated, and to inform neuropsychological and scholastic assessments
**NP2. Neuropsychological deficits are common in TSC, even in those with normal intellectual ability, and should be screened for.** Specific neuropsychological deficits (in brain-referenced systems such as memory, attentional and executive skills, language, and visuospatial skills) are very common in TSC and are seen even in those with high to very high intellectual ability. For individuals with normal intellectual ability, neuropsychological deficits are seen across age, sex, and genotype (*TSC1* or *TSC2*), and in those with and without epilepsy. Neuropsychological skills (and deficits) emerge during neurodevelopment and may not always be present at the time of a TSC diagnosis. For this reason, children and adults with TSC should have ongoing screening and monitoring for the presence or emergence of neuropsychological deficits
**NP3. Neuropsychological deficits can have a major impact on the functional ability of an individual and may present in various ways.** The presence of specific neuropsychological deficits may manifest in many different ways (e.g. anxiety and feeling overwhelmed, dysregulated behaviors, difficulties in education, or struggles in work). The manifestations may present differently based on the age and developmental level of the individual and on the specific neuropsychological deficit. Caregivers and healthcare providers should therefore establish how these deficits manifest in daily life. This may help understand current challenges, anticipate future difficulties, and inform interventions or support
**NP4. Individuals with TSC who are known to have a diagnosed neurodevelopmental disorder (such as ADHD, autism, and known learning disorders, e.g., reading/writing/maths) should receive a formal neuropsychological evaluation for the presence of neuropsychological deficits.** Even though all people with TSC are at risk of neuropsychological deficits, those with neurodevelopmental disorders such as ADHD, autism, and learning disorders are particularly likely to have specific neuropsychological deficits. For this reason, these individuals should not only receive screening for neuropsychological deficits but should be referred for formal evaluation. Neuropsychological evaluation may identify a particular profile of strengths and weaknesses to guide intervention, support, and accommodations
**NP5. There are non-pharmacological coaching and training strategies that can be used to strengthen areas of neuropsychological deficits.** There is an increasing body of evidence supporting a range of coaching and training strategies to strengthen or enhance specific neuropsychological skills (e.g., executive coaching for working memory, cognitive flexibility, or planning deficits). Caregivers and professionals are encouraged to explore such options when neuropsychological deficits are identified
**NP6. Neuropsychological deficits may require specific accommodations or supports in education and/or the workplace.** Accommodations or supports that are specifically designed for deficits in memory, executive skills, visuospatial, and language skills should be incorporated into individual educational plans (IEPs) or equivalent special educational frameworks and should also be considered in the workplace for adults

For these reasons, the consensus panel recommended that all individuals with TSC should have a comprehensive assessment of their intellectual abilities (NP1) and should be monitored with annual screening for the emergence of neuropsychological deficits (NP2). The recommendations highlighted the fact that specific neuropsychological deficits may have an impact in many ways, including mood and anxiety difficulties (e.g., feeling anxious or easily overwhelmed), having dysregulated behaviors when task demands become too much (e.g., when expected to switch flexibly between tasks), or on their scholastic skills (e.g., in reading, writing, or mathematics) (NP3). Individuals with known neurodevelopmental disorders should receive a detailed evaluation of their profile of neuropsychological strengths and weaknesses and not only be screened for such deficits (NP4). Importantly, the consensus panel recommended that non-pharmacological coaching and training strategies should be used to strengthen areas of neuropsychological deficits (NP5) and that evidence of neuropsychological deficits is likely to require accommodations and specific support in educational or occupational settings (NP6).

### Overactive/impulsive cluster

The overactive/impulsive cluster recommendations are shown in Table 8. Overactive, impulsive, and restless behaviors are very common in TSC. Even though the manifestations are typically lower in adults than children, they are seen across ages, sex, and different genotypes [14, 29]. There are many possible reasons why people with TSC have difficulties in this cluster, including physical health, developmental, or environmental reasons. However, a significant proportion of people with difficulties in this cluster may meet the criteria for ADHD and may therefore benefit from the evidence-based treatment strategies for ADHD as recommended in the general population [45], even when they may have co-occurring seizures [46], autism [33], and/or intellectual disability [47, 48].

**Table 8:** Overactive/impulsive cluster recommendations

**OI1. Overactive and impulsive behaviors are common in TSC and should be evaluated in children and adults.** It is common to see overactivity, impulsivity, and restless behaviors in TSC. Even though manifestations typically have lower rates in adulthood than childhood, it may be seen across age, developmental level, sex, and genotype (*TSC1* or *TSC2*). It is therefore important to screen for these behaviors on a regular basis and to proceed to the next steps when identified
**OI2. All individuals with overactive and impulsive manifestations should be considered for a clinical diagnostic assessment for attention deficit hyperactivity disorder (ADHD).** There may be many different reasons why individuals with TSC present with overactivity, restlessness, or impulsivity. These may include physical health (e.g., manifestations of physical illness or adverse effects of medications), developmental (e.g., in keeping with the developmental level of an individual), environmental (e.g., an overstimulating environment), or mental health reasons (e.g., as part of a psychiatric disorder). ADHD is the most common psychiatric disorder associated with overactive and impulsive behaviors. However, it is also important to consider other psychiatric disorders that may be associated with overactive and impulsive behaviors (e.g., anxiety disorders, autism, or impulse control disorders). To make a reliable psychiatric diagnosis requires training and expertise. Whenever possible, evaluation and diagnosis should be made by a specialist clinician trained in psychiatric disorders
**OI3. ADHD in TSC should be diagnosed and treated using the evidence-based approaches and intervention guidelines in the general population.** There are international guidelines for the diagnosis and treatment of ADHD in the general population that are based on good evidence and expert consensus. In the absence of a TSC-specific evidence base, these guidelines should be used, whilst maintaining a mindful approach to the physical and TAND-related complexity of TSC
**OI4. When an individual with TSC has moderate-severe ADHD, treatment with methylphenidate or other stimulant medications should be considered.** The core manifestations of ADHD include inattention, overactivity, and impulsivity. When these are associated with a diagnosis of moderate-severe ADHD, healthcare providers should consider the use of stimulant medications. Despite the theoretical concern of stimulant medications in relation to seizures in TSC, this is not the clinical experience of the consensus panel, and treatment with methylphenidate or other stimulant medications is therefore recommended for moderate-severe ADHD. Importantly, the medical treatment of ADHD should always be embedded into a comprehensive “bio-psycho-social” treatment plan
**OI5. Even when ADHD is accompanied with epilepsy, intellectual disability, autism, or other physical or TAND manifestations, ADHD symptoms may respond appropriately to treatment.** Acknowledging the high likelihood of co-occurring physical health and TAND manifestations in TSC, appropriate treatment of ADHD with evidence-based treatments as for ADHD in in the general population should be considered
**OI6. Further research is required to improve the understanding of overactivity, restlessness, and impulsivity in TSC.** There is a remarkable gap in the research literature concerning overactivity, restlessness, and impulsivity in TSC, in spite of the high prevalence rates of these behaviors. Further research is required to expand this knowledge base, including (but not limited to) evaluating non-pharmacological and pharmacological interventions for overactive and impulsive behaviors and ADHD in TSC

For these reasons, the consensus panel recommended ongoing screening for difficulties in this cluster and to proceed to appropriate next-step evaluations when difficulties are identified (OI1). All individuals who have difficulties in this cluster should be considered for a diagnostic assessment for ADHD (OI2) and, if diagnosed, be treated using the evidence-based approaches as recommended in the general population (OI3). When ADHD manifestations are moderate-to-severe, treatment with methylphenidate or other stimulant medications should be considered alongside non-pharmacological strategies to support the individual (OI4). Even when ADHD is accompanied by epilepsy, intellectual disability, autism, or other physical or TAND manifestations, the ADHD symptoms may respond appropriately to pharmacological treatments (OI5). The consensus panel also recommended further research to understanding pathways to overactive and impulsive behaviors and to generate an evidence base for intervention strategies (pharmacological and non-pharmacological) for these manifestations in TSC (OI6).

### Scholastic cluster

The scholastic cluster recommendations are shown in Table 9. Scholastic difficulties are very common in TSC regardless of the intellectual abilities of individuals with TSC, with rates around 60% [15]. There are often early risk markers of later scholastic difficulties such as the delayed onset of language development, difficulties in social communication, or other developmental milestones [49–51]. The majority of school-aged children with TSC are therefore likely to benefit from additional support and/or a personalized approach to their education.

**Table 9:** Scholastic cluster recommendations

**S1. Scholastic difficulties are common in TSC and should receive early and ongoing screening, followed by appropriate action.** The rates of difficulties in scholastic skills (e.g., reading, writing, spelling, mathematics) are very high in TSC (~ 60%) and can persist into adulthood. For this reason, all children with TSC should have early and ongoing screening to identify difficulties, gaps, and concerns about scholastic skills, followed by formal assessments as needed
**S2. Delays in language development, counting, social/communication skills, and other developmental milestones may be markers for later scholastic difficulties.** Caregivers, healthcare providers, and educational teams should monitor for signs of difficulties in language development, counting and math skills, social and communication skills, and other milestones that may indicate a potential for later school difficulties
**S3. All children with TSC should be considered for an individual educational plan (IEP/IEDP) to support their learning.** Given the high rates of scholastic difficulties, all children with TSC should be considered for an appropriate individual education plan (referred to as an “IEP” or “IEDP” in many countries) or its equivalent. An IEP/IEDP is a legal document drawn up by an educational authority with the family and outlines requirements to support students who need accommodations, curriculum modifications, or alternative educational programs
**S4. School-aged children with TSC should be supported in the most appropriate educational environment to meet their needs.** Caregivers and educators should advocate for the most appropriate educational environment that would be beneficial for the child with TSC. Given the wide range of TAND profiles in TSC, an appropriate educational environment could range from a mainstream school, to a fully inclusive environment, or a “special” school or classroom. The options and programs vary significantly in different countries, and families should seek the most appropriate environment to meet their child’s needs in partnership with the relevant educational authorities
**S5. In all educational environments, educators should use high-quality teaching strategies, Response to Intervention (RTI), individualized teaching, and appropriate accommodations to ensure student success in scholastic skills (e.g., reading, writing, spelling, and math) and in all other aspects of education.** The educational needs of children with TSC are highly heterogeneous and children with TSC often present with a range of educational strengths and weaknesses that require high-quality educational support. For this reason, educators should implement a highly personalized program of teaching, accommodations, modifications, and alternatives using high-quality teaching strategies and response to intervention (RTI) approaches
**S6. Educators should monitor the overall TAND profile of each child with TSC and consider how it may affect the child’s ability to access education.** Educators should be aware of each student’s TAND profile and how it may affect their education. This may include difficulties in many TAND clusters and may be associated with specific psychiatric diagnoses (e.g., ADHD, autism, mood, and anxiety disorders). Educators should, in particular, monitor for neuropsychological deficits (particularly in executive and attentional skills) and provide appropriate supports, such as schedules, outlines, organizers, and assistance in planning and breaking down assignments into parts. Social support may also be needed, for example, support in terms of making and maintaining healthy relationships. Teachers, special education teachers, or others within the school should monitor for social difficulties and provide support and teaching, to support the development of the social skills of students. This broad view of a child’s TAND profile should lead to the incorporation of appropriate educational strategies, accommodations, and goals to support each child with TSC
**S7. Plan for educational transitions.** For all educational paths, transitions through each stage of schooling and beyond secondary education must be considered and planned for early. Educators and clinicians should suggest resources and provide information to support these important transitions

For these reasons, the consensus panel recommended early and ongoing screening for scholastic cluster difficulties followed by appropriate action when concerns are identified (S1). The panel highlighted that delays in early developmental milestones may be markers of future scholastic difficulties (S2). All children with TSC should be considered for an individual educational plan (IEP/IEDP) to support their individual profile of learning needs (S3). There is no “one-size-fits-all” in education provision for children with TSC, and the goal should therefore be to match the comprehensive needs of each child with the most appropriate educational environment (S4). Acknowledging that educational environments and supports may vary significantly across the globe, the panel recommended that in all educational settings, high-quality teaching strategies, response-to-intervention (RTI) approaches, and appropriate accommodations (e.g., differentiated reading material, seats close to the educator, extra time, quite spaces, chunked assignments) should be provided [52, 53] (S5). Educators should monitor the overall TAND profile of each child with TSC and consider how it may affect the child’s ability to access education (S6). Planning for educational transitions through each stage of schooling and beyond secondary education was also recommended (S7).

### Wraparound psychosocial cluster

The wraparound psychosocial cluster recommendations are shown in Table 10. The psychosocial health and wellbeing of individuals and families who live with TSC is a priority area, yet very little research has been conducted in this domain, and very little is typically done to evaluate and support the psychosocial needs of families [54–58].

**Table 10:** Wraparound psychosocial cluster recommendations

**PS1. Monitor the psychosocial health and wellbeing of all individuals with TSC.** TSC is associated with a very significant impact on the wellbeing of individuals. The psychosocial health and wellbeing of children, adolescents, and adults with TSC should therefore be monitored systematically, especially in those who are more severely affected by TSC manifestations. This should include considerations of self-esteem, family stress, relationship difficulties (e.g., with siblings or parents), as well as the ability to connect with others in the community or to progress in their school, work, or career. Psychosocial screening may include direct observation, standardized instruments, and reports from family members or other caregivers
**PS2. Monitor the psychosocial functioning of all family members who live with TSC.** Caregivers and family members of individuals with TSC also experience a significant burden on their health and wellbeing. For example, they are more likely to have mental health problems (e.g., depressive disorder or coping difficulties) than the general population. For this reason, the health and wellbeing of families who live with TSC should also be monitored systematically and comprehensively. This should include considerations of self-esteem, family stress, relationship difficulties (e.g., between siblings, parent–child, or parent-to-parent difficulties). In addition, very high levels of stress can lead to difficulties for families to connect with others in their community and may impede the ability of caregivers to progress in their own education, work, or career
**PS3. Provide integrated and well-coordinated care to families who live with TSC.** Pursuing integrated care with well-coordinated services could result in improved quality of care and outcomes, reduced healthcare costs, and increased family wellbeing. This type of care can be found at expert TSC networks, TSC centers, or TSC clinics. In contexts where expert TSC services or systems are not available, the appointment of one person to act as “care/clinical coordinator” (to maintain an overview of the trajectory and needs of an individual with TSC and their family and who can help to coordinate all aspects of care) is highly recommended and can have beneficial effects on health and reduce healthcare expenses
**PS4. Comprehensive family-centered care should include psychosocial interventions and practical support.** Where individuals with TSC and/or their family members have psychosocial needs, practical supports, and psychosocial interventions (e.g., psychological therapies) should be provided. For example, when high levels of stress are observed in the family, service providers should assess the psychological and social needs of families and help them with targeted supports that could help to reduce stressors (e.g., provision of care support, respite or relationship support) and tools to increase psychological resilience (e.g., through psychological support)
**PS5. Focus on and measure the quality of life of individuals with TSC and their families.** A well-coordinated, multi-disciplinary approach to the management of TSC can improve the quality of life of individuals and caregivers who live with TSC. Individual and family quality of life should therefore be monitored informally and using standardized tools. Higher levels of quality of life are associated with increased social interests and active participation, less negative feelings and concerns, and fewer restrictions on physical activities
**PS6. Provide dedicated support to individuals and caregivers to optimize their employment and professional lives.** The employment and professional lives of those with TSC and their caregivers are likely to be affected by TSC, as evidenced by reduced work productivity, increased absenteeism, and higher levels of impairment in their daily life activities. Young individuals with TSC should be supported with access to facilities providing study guidance. Adults with TSC should have access to career counseling, assistance seeking work, and coaching at work. For caregivers, this may include discussions about the option to reduce working hours or workload, or finding alternative financial support to allow caregivers to provide optimal assistance to their family while maintaining an optimal work/life balance
**PS7. Care for the caregivers.** Given the impact of TAND on individuals and caregivers, and given the fundamental role of caregivers and families as expert partners in the life journey of people with TSC, supporting and empowering caregivers and ensuring their wellbeing is paramount. Individuals with TSC can only achieve optimal outcomes if we also look after the wellbeing of those who care for them. Careful monitoring of caregiver quality of life (including using standardized measurements) and dedicated time during clinical consultations to discuss family and caregiver wellbeing can have significant positive effects on family quality of life

For this reason, the consensus panel recommended monitoring of the psychosocial health and wellbeing of all individuals with TSC (PS1) and of all their family members (PS2), using screening (e.g., with the TAND-SQ Checklist), observation, family reports, or other standardized instruments. Based on their psychosocial needs, families should be provided with integrated and well-coordinated care (PS3). This should include practical support as well as psychosocial interventions (e.g., psychological therapies) as required (PS4). The overall focus should be on “family quality of life” by helping individuals and families identify their goals towards, for example, social activities and active participation (PS5). TSC often has a major impact on the employment and professional lives of individuals with TSC and their families. This should therefore also be a specific focus of psychosocial support provided (PS6). The consensus panel recognized that families and caregivers are paramount to the wellbeing of individuals with TSC and of the whole family. It is therefore of fundamental importance to “care for caregivers” by monitoring caregiver wellbeing, dedicating time in consultations to family and caregiver wellbeing, and generating evidence of interventional approaches that could strengthen caregiver wellbeing (PS7).

## Discussion

In this study, we set out to generate evidence-informed consensus recommendations for the identification and treatment of TSC-Associated Neuropsychiatric Disorders (TAND). We used a highly systematic process for evidence evaluation and consensus building. The process led to a set of ten core principles, seven sets of cluster-based clinical recommendations, and recommendations for a “wraparound” psychosocial cluster. Recognizing that individuals and families with TSC live in highly diverse global contexts and health, educational, and social care systems, we prioritized conceptual (“big picture”) recommendations over highly detailed ones.

As outlined in the introduction, clinical practice recommendations for TAND over the years have moved from assessment and treatment when clinical concern was observed (1999), to comprehensive assessment at key developmental timepoints (2005), to annual screening (2013), and to early identification and treatment and psychosocial support for families (2021). The novel contribution of the consensus recommendations presented here was the focus on TAND clusters with the cluster-based evidence review and consensus-building. The search for natural TAND clusters started in response to the “overwhelming uniqueness” of the TAND profiles of individuals described by families and TSC clinicians which made them experience a “treatment paralysis” [11]. Data-driven methodologies using TAND-L Checklist data showed that 7 natural clusters of TAND could be identified [19–21]. It is important to observe that, although these clusters had good internal consistency and were therefore coherent within themselves, there is clearly significant overlap and co-occurrence between clusters. For example, the autism-like cluster very often co-occurs with characteristics of other clusters. This observation is summarized in core principle #5 which reminds us that clusters cluster together. Even though the clinical reality is therefore co-occurrence of cluster manifestations, the value of cluster-based identification and treatment lies in the fact that the potentially very complex TAND presentation of an individual can be divided into more manageable chunks for which diagnostic and treatment options may exist.

**Fig. 3:**
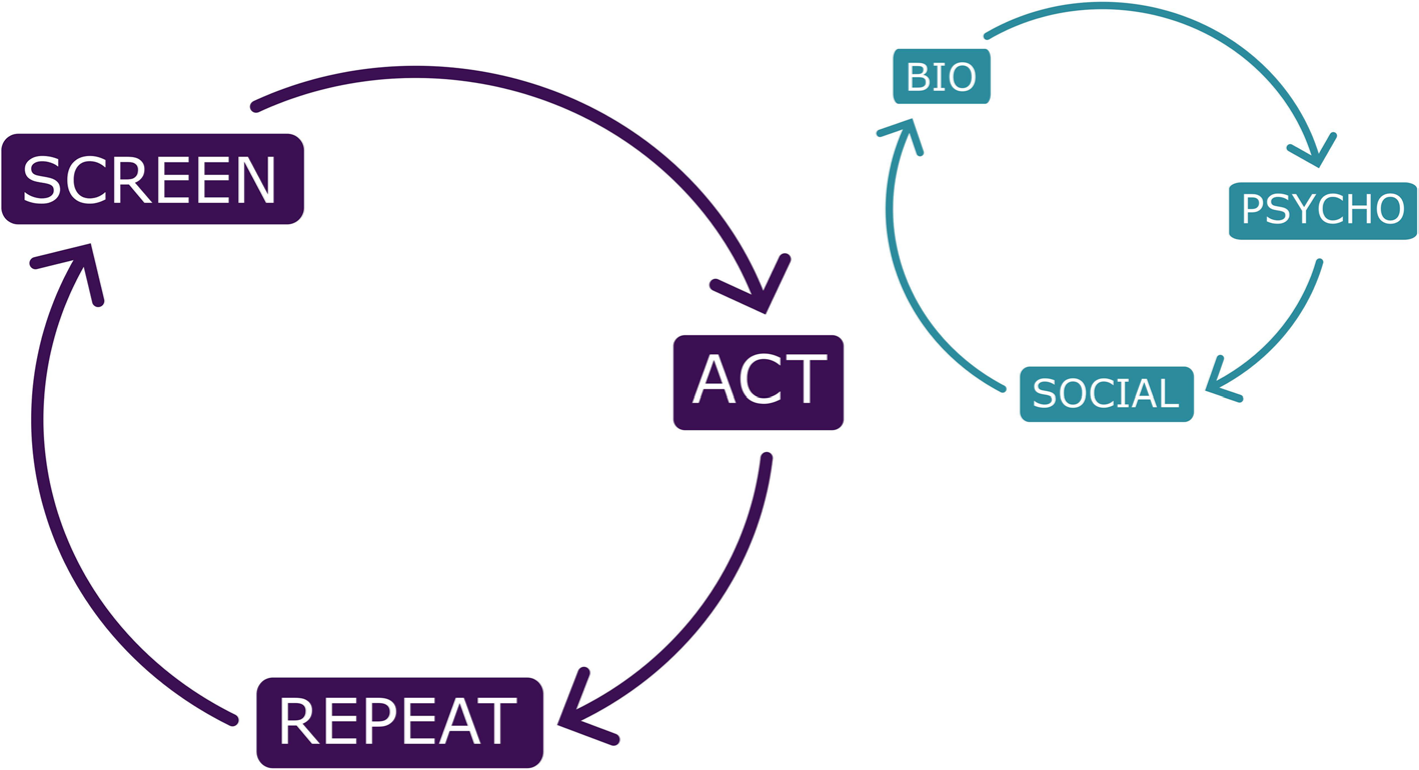
Overarching recommendation for identification and treatment of TAND

These consensus clinical recommendations represent the first systematic approach to the identification and treatment of TAND. Taking together all the recommendations presented, the overarching recommendation is shown in Fig. 3 and can be summarized in three words—screen, act, repeat. Caregivers and their support teams in health, social care, and education are encouraged to “screen” for TAND at least annually using screening tools such as the TAND-L or TAND-SQ Checklists [5, 59]. Screening in this context refers to a systematic topline check to identify any existing or emerging concerns in the individual with TSC and/or their caregiver system. Screening can be performed by anyone with appropriate clinical expertise or by caregivers themselves, but should ideally be done in participation with a TSC clinic or other relevant clinical team who can support the family.

Where screening identifies any concerns, this should be followed by action (referring to “act” in Fig. 3). Action should include further detailed evaluations (e.g., of development, for specific disorders, of educational profile and needs, of psychological profile and needs, and of social needs) to inform the most appropriate intervention or treatment plan. Action should be broad and should include a bio-psycho-social approach, where all relevant biological, psychological, and social contributors (and their treatments) are considered and integrated.

Once appropriate action has been taken, it is important to “repeat” the process at least annually to ensure that new and emerging concerns are identified and acted on as soon as possible.

Following the generation of these clinical recommendations, the next important task will be engagement with the international TSC community to ensure the appropriate translation and implementation of these new recommendations across the globe. Targeted dissemination and implementation will need to involve family stakeholder groups (such as Tuberous Sclerosis International and its strong network of national TSC organizations), as well as professional partners in health, social care, and educational settings. One of the next step strategies identified as a specific objective in the TANDem project is to use the consensus recommendations as the foundation for the creation of a ‘TAND toolkit’ of information and tips on “what to seek” (e.g., further evaluations or professional support) and “what to do” (e.g., self-help strategies) to be built into a “TAND toolkit app” (see [9] for further details).

During the final review of these recommendations by the TAND consortium, we reflected on two additional elements, not captured in the core principles or cluster recommendations. The first was the recognition that children and adults with TSC, particularly those with co-occurring intellectual disability, are a vulnerable group at increased risk of abuse and neglect and that all professionals supporting TSC families should be vigilant to identify potential markers of concern [60–62]. Apart from their vulnerability to abuse and neglect, individuals with TSC should also be enabled in all possible ways to be able to express their needs and preferences and to be involved in all decisions related to their health and care.

The second reflection was that people with TSC should not be merely defined by their challenges, difficulties, and disabilities. Instead, each person with TSC has their own skills, talents, and personality that can bring great pleasure, enrichment, and meaning to the lives of those around them. In the recently developed TAND-SQ Checklist [59], we added a specific question on “strengths” in response to feedback from within the TAND consortium and TSC community, very much in keeping with the need to balance “difficulties” and “strengths.” These clinical recommendations presented here should therefore be seen as an attempt to give guidance to individuals and families who are struggling with particular aspects of their TAND profile, not to “change” or “cure” the individual, but to improve their quality of life and active participation in society.

## Conclusions

Here we presented the first set of evidence-informed consensus recommendations for the identification and treatment of TSC-associated neuropsychiatric disorders (TAND). The next steps should include participation with the broader TSC community to ensure targeted dissemination and implementation of these recommendations. We acknowledge that services and access to interventions are highly variable across the globe and that many families may not immediately be able to access some of the evidence-informed recommendations made here. However, these consensus recommendations are, in part, also presented as an aspirational set of next steps. Families and their clinical teams should therefore use these recommendations to think about what is available in their local communities and collaboratively consider how to get access to support in line with the recommendations made here. We hope that these international consensus recommendations will empower families and professionals who support them with a systematic framework that will reduce the “assessment and treatment gap” in TAND across the globe.

## Data Availability

All data generated and analyzed for this paper are included in this publication and its supplementary information files.
